# Impaired renal functions in Pakistani cohort of rheumatoid arthritis

**DOI:** 10.12669/pjms.35.4.564

**Published:** 2019

**Authors:** Abrar Ahmed Wagan, Sadia Nasir, Abdul Rahim, Daim Khan

**Affiliations:** 1 Dr. Abrar Ahmed Wagan, MBBS, FCPS (Medicine), FCPS (Rheumatology), FACR. Assistant Professor of Medicine, Department of Medicine, Central Park Medical College Lahore, Pakistan; 2 Dr. Sadia Nasir, MBBS, MRCP (UK). Assistant Professor of Medicine, Department of Medicine, Central Park Medical College Lahore, Pakistan; 3 Dr. Abdul Rahim, MBBS. Postgraduate Trainee, Department of Medicine, Central Park Medical College Lahore, Pakistan; 4 Dr. Daim Khan, MBBS. Postgraduate Trainee, Department of Medicine, Central Park Medical College Lahore, Pakistan

**Keywords:** DMARDS’s, Estimated Glomerular filtration rate, Hypertension, Impaired renal function, Rheumatoid arthritis

## Abstract

**Objective:**

To determine the frequency of impaired renal functions and hypertension in rheumatoid arthritis.

**Methods:**

This study was conducted between May 1^st^ 2018 to February 1^st^ 2019 at Rheumatology Division, Department of Medicine Central Park Medical College Lahore, total 260 study participants were selected, demographic detail were asked in detail, disease duration of RA and hypertension, DMARD’s, self-use NSAID’s,/hakeem medications, smoking were asked in detail, BMI and blood pressure were measured,5 ml of blood was taken by trained phlebotomist, and sent for the estimation of serum urea and creatinine on (COBAS-III) machine, after availability of results each individuals eGFR (creatinine clearance) was calculated by Cockroft Gualt_(CG)_ and Modification in diet in renal disease method (MDRD).

**Results:**

In this study the mean age of study participants was 42.4 (± 9.5) years with disease duration of 7.7(±4.8) years, prevalence of Impaired renal functions of 14.6% (n=38) and hypertension in 53.5% (n=139).Regression analysis shows there is significant association between hypertension, smoking and self/hakeem medications with impaired renal functions (p-0.5). Kappa analysis shows both (MDRD & CG methods) had uniformity in picking up cases of impaired renal functions 75.6% (p-0.05).

**Conclusion:**

In RA decline in renal functions is seen with self-use NSAID’s/hakeem medications along with other modifiable factors like smoking and hypertension, while conventional DMARD’s don’t show association with decline. There is very high prevalence of hypertension in rheumatoid arthritis.

## INTRODUCTION

Rheumatoid arthritis as common inflammatory arthritis affects 0.5 to 1% population worldwide.^1^ In Canadian study it was found that these patients had 17 years shorter median survival than general population, due to increased prevalence of comorbidities like myocardial infarction, stroke and heart failure.^2^ In RA estimated prevalence of hypertension was (70.5%).^3^

It can be explained by several factors like persistent systemic and low-grade inflammation, which leads to hypertension via several mechanisms, reduction of nitric oxide production in endothelial cells leading to vasoconstriction, increased production of endothelin-1, and platelet activation. Moreover, CRP is able to up-regulate the expression of angiotensin Type-I (AT1) receptors thus activating the renin-angiotensin system (RAS), oxidative stress, increased total peripheral resistance, medications, lack of physical activity, environmental factors and above all genetic polymorphism conferring increased risk.^3^-^5^ With current treatment modalities like anti-tumor necrosis factor and anti–interleukin 6 biologics appear to have beneficial effects on endothelial function and arterial stiffness and thus may reduce blood pressure independently of disease activity.^6^-^8^

The National Health Survey of Pakistan estimated that hypertension affects 18% of adults and 33% of adults above 45 years, and alarmingly only 50% of the those people were diagnosed and only half of those were treated.^9^ In COMEDRA French nationwide cross-sectional multicenter study on comorbidities in RA, renal functions were assessed from the estimated glomerular filtration rate (eGFR), using the Modification of Diet in Renal Disease equation, it was found (8.8%) patients with RA had impaired eGFR and (9%) had proteinuria.^10^

RA patients with renal disease were found to have significantly increased mortality, to those with normal renal functions, HR Ratio (2.77–4.45).^11^ Autopsy findings in RA has shown that in (3–20%) cases renal failure was a major cause of deaths.^12^,^13^ Renal decline in RA is clinically important because it not only restricts the management of primary disease, but also increases mortality so regular assessment of renal function has pivotal importance in long term disease control.

## METHODS

After the approval of ethical review committee, this cross sectional study was conducted in outpatient department of Rheumatology division of Medicine at Central Park Medical College Hospital Lahore. Written and informed consent were taken from each participant. RA was diagnosed based on 2010 ACR criteria. Sample size was calculated using OpenEpi sample size calculator, by inserting 8.8% prevalence of impaired renal functions and 3.45% margin of error with 95% confidence interval n = 260.^10^

Rheumatoid arthritis participants who were seropositive for either or both (Rheumatoid factor and ACPA) were included, sero-negative rheumatoid arthritis, psoriatic arthritis, systemic lupus erythematous, scleroderma, primary osteoarthritis, mixed connective tissue diseases, current or past six months use of biological disease modifying drugs, known cases of heart failure, myocardial infarction in last month, hemodialysis, diuretics use, known cases of chronic liver diseases, plasmaphresis in last two months, impaired cognition, history of organ transplant in last two years and using calicneurin inhibitors, history of chemotherapy or radiation therapy in past two years, patients who were admitted in hospitals and diagnosed as acute renal failure due to hypovolemic/septic shock in past three months were excluded from study.

Demographic details were noted for each study participant, with RA duration, number and duration of medicines in use, smoking status, history of self-use NSAID’s/hakeem medication, use of corticosteroid were inquired in detail. Hypertension was defined as systolic blood pressure ≥140 mm Hg or diastolic blood pressure ≥90 mm Hg on two last readings five minutes apart and after five mandatory rest, or currently taking antihypertensive medicine, basal metabolic index was calculated by standardized formula, afterward 5 ml of blood was taken by a trained phlebotomist for serum urea and creatinine measurement with BD syringe then sampled was analyzed on (COBS-III) machine.

Two methods were used to estimate renal function: the Cockcroft–Gault formula for creatinine clearance in ml/min ((140 − age in years) × body weight in kg/ (serum creatinine in μmol/l × 0.81) × 0.85 if female); and Modification of Diet in Renal Disease (MDRD) formula for glomerular filtration rate (GFR) in ml/min/1.73 m2 (186.3 × (age in years (−0.203)) × serum creatinine in μmol/l / (88.4(−1.154)). MDRD formula was the primary measure, as it is the most reliable in patients with decreased renal function and is currently the most widely used formula for estimating renal function in clinical settings.

All study participants were examined in details by senior consultant level physician for health evaluation. Data was stored and analyzed using IBM-SPSS version 23.0, count and percentages were reported for prevalence of hypertension and impaired renal function based on MDRD, association of these two outcomes was tested with age group, gender, body mass index and other qualitative studied parameters, association of outcomes for impaired renal function based on MDRD and creatanine clearance was tested using Kappa test of agreement, all p-values less than 0.05 were considered significant, bar chart also used to present the prevalence of study outcomes.

## RESULTS

In this study the mean age of study participants was 42.4± (9.5) years, disease duration of 7.7(±4.8) years, Body Mass Index was 27.69 (±5.7), mean systolic blood pressure 127.8 (±16.4) and diastolic blood pressure was 82.8 (±8.6), eGFR measured CG method was 112.02/ml/min and with Modification in diet in renal disease method was 92.09/ml/min (±35.1) Table-I.

**Table I:** Descriptive statistics of studied parameters.

Parameters	Mean	S.D
Age (years)	42.43	9.51
Body Mass Index (kg/m^2^)	27.69	5.73
SBP	127.78	16.40
DBP	82.80	8.63
Duration of Hypertension	2.21	2.90
Serum Creatinine	0.88	0.33
eGFR (CG method)(ml/min)	112.02	41.13
eGFR (MDRD)	92.09	35.19
RA Disease Duration	7.74	4.81

The prevalence of Impaired renal functions of n=38 (14.6%) by MDRD method and hypertension in n=139 (53.5%) as shown in Figure-I. The association of studied factors with impaired renal functions (MDRD) n=38 (14.6%) participants had impaired functions and n=36 (94.7%) were aged more than >45 years p-0.01, female gender was predominant n=29 (76.3%) males n=9 (23.7%) p-0.87 are shown in Table-II.

**Fig. 1:**
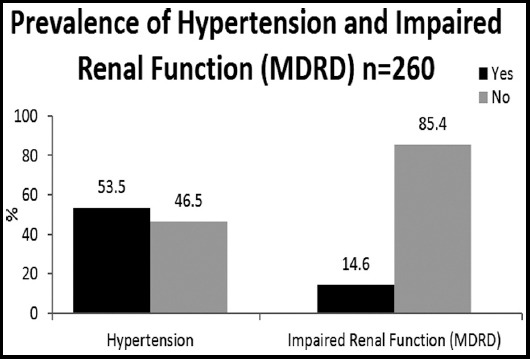
Prevalence of Impaired Renal Functions and Hypertension.

**Table II:** Association of studied factors with impaired renal function (MDRD).

Characteristics		Impaired Renal function (MDRD)				p-value

		No		Yes		

		n	%	n	%	
Age Group	24- 34 years	59	26.6	0	0.0	<0.01*
	35- 45 years	98	44.1	2	5.3	
	>45 years	65	29.3	36	94.7	
Gender	Male	50	22.5	9	23.7	0.87
	Female	172	77.5	29	76.3	
BMI (kg/m2)	≤30	153	68.9	30	78.9	0.21
	>30	69	31.1	8	21.1	
HTN on Medicine	Yes	72	32.4	20	52.6	<0.01*
	No	150	67.6	18	47.4	
Duration of HTN	1-3 years	50	47.6	10	34.5	<0.01*
	4-6 years	44	41.9	8	27.6	
	>6 years	11	10.5	11	37.9	
Number of Medicine	one medicine	40	54.8	9	45.0	0.43
	more than one medicine	33	45.2	11	55.0	
Smoking	Yes	38	17.1	12	31.6	0.03*
	No	184	82.9	26	68.4	
DMARDS	MTX	109	49.1	13	34.2	0.22
	SSZ	33	14.9	5	13.2	
	MTX COMBINATION	65	29.3	14	36.8	
	NON MTX	4	1.8	2	5.3	
	NO DMARDS	11	5.0	4	10.5	
Disease Duration	1-3 years	52	23.4	0	0.0	<0.01*
	4-6 years	61	27.5	8	21.1	
	>6 years	109	49.1	30	78.9	
HAKEEM/HOMEO/self/Nsaids	Yes	64	28.8	28	73.7	<0.01*
	No	158	71.2	10	26.3	

* p<0.05 was considered significant using Pearson Chi Square test of Independence

BMI of <30 was n=30 (78.9%) and BMI >30 n=8 (21.1%) p-0.21, while mostly were on antihypertensive medications (n=20 (52.6%) p-0.01, smoker n=12 (31.6%) p-0.03. Methotrexate alone n=34 (13.2%) and in combination was used by n=14 (36.8%) p-0.22. Longer duration diseases showed significant association with impaired renal functions p-0.01.Self-use NSAID’s/hakeem medications was significantly associated with declining renal functions p-0.01.

Odds ratio with 95% confidence interval for impaired renal functions, females are 0.94 and obese are also less likely to suffer from impaired renal functions, while hypertensive patients with duration of more than six years, smokers, and those who have history of self-use NSAID’s/hakeem medications are more likely to suffer from impaired renal functions p-<0.01. Table-III

**Table III:** Risk estimation of impaired renal function (MDRD) using binary logistics regression analysis.

Characteristics		Impaired Renal function (MDRD)	p-value

		Odds Ratio (95% C.I)
Gender	Male	Reference	0.87
	Female	0.94(0.42-2.11)	
BMI (kg/m2)	≤30	Reference	0.21
	>30	0.6(0.26-1.36)	
Hypertension	No	Reference	<0.01*
	Yes	3.89(1.71-8.86)	
Duration of HTN	1-3 years	Reference	
	4-6 years	0.91(0.33-2.51)	0.84
	>6 years	5(1.71-14.68)	<0.01*
Smoking	No	Reference	0.04*
	Yes	2.24(1.04-4.82)	
DMARDS	MTX	0.33(0.1-1.19)	0.08
	SSZ	0.42(0.1-1.84)	0.24
	MTX COMBINATION	0.6(0.17-2.14)	0.42
	NON MTX	1.38(0.18-10.66)	0.76
	NO DMARDS	Reference	
HAKEEM/HOMEO/self/Nsaids	Yes	0.15(0.07-0.32)	<0.01*
	No	Reference	

* odds ratio considered significant with p-value less than 0.05

Cohen kappa analysis show two renal function measures MDRD and CG method for renal functions, results showed 28 (10.76%) out of 38 cases were commonly declared as positive, while 217 (83.4%) cases were commonly declared as negative, there was (75.6%) agreement obtained between two measures for impaired renal functions (p-0.5). Table-IV

**Table IV:** Association of cretanine clearance and MDRD impaired renal function using Kappa analysis.

Measures		Impaired Renal function (MDRD)		Total

		No	Yes
Impaired Renal function (Cretanine Clearance)	No	217	10	227
	Yes	5	28	33

Total		222	38	260

Kappa = 75.6%, P<0.01.

## DISCUSSION

In rheumatoid arthritis renal system is jeopardized by diseases process and the medicines for control, and sometimes it becomes really difficult to differentiate to know the cause of impairment in renal functions. Renal involvement not only seriously undermines the efforts to treat the RA but also increases the morbidity and mortality by increasing the cardiovascular events. The CARRÉ Study a prospective cohort of patients with RA, in whom CV events and concurrent risk factors were investigated, 353 RA cases were followed for three years and results showed, patients who had CV event had a significantly lower baseline GFR than those who did not (59 vs 79 ml/min, p=0.001), this association remained significant after adjustment for traditional risk factors, decrease in GFR of 5 ml/min was associated with a 30% (95% CI 7% to 59%) increase in the occurrence of CV events.^14^

Study by Hsien-Yi C et al., RA patients with concurrent CKD had significantly higher likelihood of developing ischemic heart disease and stroke, after adjusting for traditional cardiovascular risk factors, it was independently associated with a significantly increased risk of CKD (adjusted hazard ratio 1.31; 95% confidence interval 1.23–1.40) and glomerulonephritis (aHR 1.55; 95% CI 1.37–1.76).^15^

In MATRIX study (MeThotreXate and Renal Insufficiency), of 129 patients, 102 (79.1%) patients had available data for Serum Creatinine, 19 of them (18.6%) had an abnormal Serum Creatinine levels.^16^

In our study prevalence of impaired renal functions was (14.8%) with lower mean age and short RA duration while in COMEDRA French study it was (8.8%).^10^ RA patients who had undergone kidney biopsy, findings were mesangial proliferative glomerulonephritis, membranous nephropathy, IgA nephropathy, minimal change disease, pauci-immune glomerulonephritis, analgesic nephropathy, interstitial nephritis, and AA amyloidosis.^17^,^18^ In a recent study, excess weight was associated with CKD in RA.^19^ While in our study we couldn’t find association between obesity and decline in renal functions, Hsien-Yi C et al.^15^ also reported same.

Daoussis D et al. found that female sex, advanced age, increased serum uric acid levels, the presence of extra-articular disease, and increased cholesterol levels were independently associated with decreased kidney function in a cross sectional, single-center study of 400 consecutive patients with RA.^20^ Masako K et al., retrospective study found persistently high CRP as an independent predictor of the incidence of CKD (hazard ratio 3.00; 95% confidence interval, 1.23–8.53; P = 0.01).^21^

Regression analysis shows significant association of smoking, self-used NSAID’s/ hakeem medications and longer duration of hypertension as risk factor for development of impaired renal functions (p-<0.5). Shunsuke M et al., found female sex, obesity, hypertension were independently associated with absolute risk of eGFR based renal function decline.^22^ As the management of RA has taken a big leap forward from conventional DMARD’s to biological DMARD’s, their use is ever increasing, Keiichi S et al., in very recently published study found that use of biological DMARD’s is not associated with incident CKD and continuous decline in eGFR.^23^

We found that hypertension is widely prevalent in our RA patients (53.5%) and it was associated with decline in renal functions but at younger age in comparison to other studies, Panoulas VF,^3^ in multivariate logistic regression analysis noted that age, prednisolone use, BMI were independently associated hypertension. Hypertension also remains the neglected aspect of holistic treatment plans of RA patients and in old age group compliance is not followed strictly.

### Limitations of the study

Our study has few limitations like it was a cross sectional study, sample size was small, but this has few strong points, like this is one of the first study carried out on local population about renal functions of RA patients and hypertension prevalence, we took effects of self-used NSAID’s /hakeem medications on renal functions.

## CONCLUSION

RA being the chronic inflammatory disease has long lasting devastating effects driven by chronic inflammation due to its mediators if remains untreated, one of the major organs affected directly and indirectly are kidneys. As such in clinical practice its absolutely mandatory to check renal functions periodically in resource limited third world countries, awareness campaigns are needed to inform public about deleterious effects of hakeem medications/self-medications, hypertension with other modifiable cardiovascular risk factors kept in well control, patients should be encouraged to adopt healthy life styles, with effective compliance of medications.

### Author`s Contributions

**AAW:** Conceived, designed, statistical analysis, manuscript writing, editing.

**SN:** Data collection, statistical analysis, editing, critical review.

**AR and DK:** Data collection, manuscript writing, editing.
